# Association between home food preparation skills and behaviour, and consumption of ultra-processed foods: Cross-sectional analysis of the UK National Diet and nutrition survey (2008–2009)

**DOI:** 10.1186/s12966-017-0524-9

**Published:** 2017-05-23

**Authors:** Matthew Chak Leung Lam, Jean Adams

**Affiliations:** 1 Centre for Diet & Activity Research, MRC Epidemiology Unit, University of Cambridge, Cambridge, UK; 2 Li Ka Shing Faculty of Medicine, University of Hong Kong, Pok Fu Lam, Hong Kong

**Keywords:** Cooking, Diet, Nutrition, Processed foods

## Abstract

**Background:**

‘Ultra-processed foods’ (UPF) have been industrially processed and tend to be higher in saturated fat, sodium and sugar than other foods. There is some evidence that consumption of UPF is associated with overweight, obesity and related diseases. In developed countries more than half of dietary energy is attributed to UPF. One reason for reliance on UPF may be poor home food preparation skills or infrequent use of these. This relationship has been previously proposed but not tested. We examined the relationship between home food preparation skills and behaviour and consumption of UPF.

**Methods:**

We used data from adults in the UK National Diet & Nutrition Survey 2008–09. Home food preparation skills and behaviours of adults (*n* = 509) were assessed using questions on confidence using eight cooking techniques, confidence cooking 10 foods, ability to prepare a cake or biscuits without help, and whether or not participants prepared a main meal five or more days per week. Individuals’ UPF consumption was determined from four-day estimated diet diaries. Associations were adjusted for age, gender, occupational social class and household composition.

**Results:**

In fully adjusted models, individuals who were confident with all 10 foods (adjusted beta (95% CI) = −3.76 (−6.02 to −1.50)), able to bake cakes or biscuits without help (−3.87 (−6.62 to −1.12)), and cooked a main meal at least five days a week (−2.84 (−5.43 to −0.24)) consumed a lower percentage of dietary energy from UPF.

**Conclusions:**

In UK adults better home food preparation skills and more frequent use of these skills tended to be cross-sectionally associated with lower UPF consumption. Greater encouragement of these skills may help reduce reliance on UPF.

## Background

‘Ultra-processed foods’ (UPF) are foods that have been industrially processed. [1] These foods tend to be higher in saturated fat, sodium and sugar than other foods. [2–6] Diets high in UPF tend to be lower in fruit and vegetables and fibre; and higher in sodium, saturated fat and sugar compared to diets lower in UPF. [2, 5, 7] Some, but not all, [2, 8] studies have reported positive associations between consumption of UPF and risk of overweight, obesity and markers of metabolic syndrome. [9–14].

Consumption of UPF varies internationally. In developed countries more than half of dietary energy has been attributed to UPF. [2, 3, 5, 6, 15, 16] Consumption is currently lower in less developed countries (where these foods are relatively less affordable [17]). [9, 18] Trends towards increasing consumption over time in less developed countries [10, 16, 19, 20] indicate that reliance on UPF may be a hallmark of the ‘nutrition transition’. [21].

The term ‘UPF’ was first coined as part of a classification system that assigns all foods to one of three groups based on the degree of industrial processing involved: minimally processed foods, processed ingredients and UPF (see Table 1). [1] Like UPF, processed ingredients also tend to be high in fat, salt and sugar; but diets high in processed ingredients do not. [2, 5] This may be because diets high in processed ingredients also tend to be high in minimally processed foods [5, 22] – which have the ‘healthiest’ nutritional profile of all three groups. [2–6] It has been argued that processed ingredients and minimally processed foods are key components of food prepared at home, and that high consumption of UPF indicates low consumption of food prepared at home.

**Table 1:** Food classification based on the extent and purpose of industrial processing; from [1]

Groups	Definitions	Examples
Group 1: Unprocessed or minimally processed foods	No processing or mostly physical processes used to make single whole foods more durable, accessible, convenient, palatable or safe	Fresh, chilled, frozen, vacuum-packed fruits, vegetables, fungi, roots and tubers; grains (cereals) in general; fresh, frozen and dried beans and other pulses (legumes); dried fruits and 100% unsweetened fruit juices; unsalted nuts and seeds; fresh, dried, chilled, frozen meats, poultry and fish; fresh and pasteurized milk, fermented milk such as plain yoghurt; eggs; teas, coffee, herb infusions, tap water, bottled spring water
Group 2: Processed ingredients	Extraction and purification of components of single whole foods, resulting in producing ingredients used in the preparation and cooking of dishes and meals made up from Group 1 foods in homes or traditional restaurants, or else in the formulation by manufacturers of Group 3 foods	Vegetable oils, margarine, butter, milk cream lard; sugar, sweeteners in general; salt; starches, flours, and “raw” pastas and noodles (made from flour with the addition only of water); and food industry ingredients usually not sold to consumers as such, including high fructose corn syrup, lactose, milk and soy proteins, gums, and preservatives and cosmetic additives
Group 3: Ultra-processed food products	Processing of a mix of Group 2 ingredients and Group 1 foodstuffs in order to create durable, accessible, convenient, and palatable ready-to-eat or to-heat food products liable to be consumed as snacks or desserts or to replace home-prepared dishes	Breads, biscuits (cookies), cakes and pastries; ice cream; jams (preserves); fruits canned in syrup; chocolates, confectionery (candies), cereal bars, breakfast cereals with added sugar; chips, crisps; sauces; savoury and sweet snack products; cheeses; sugared fruit and milk drinks and sugared and “no-cal” cola, and other soft drinks; frozen pasta and pizza dishes; pre-prepared meat, poultry, fish, vegetable and other “recipe” dishes; processed meat including chicken nuggets, hot dogs, sausages, burgers, fish sticks; canned or dehydrated soups, stews and pot noodle, salted, pickled, smoked or cured meat and fish; vegetables bottled or canned in brine, fish canned in oil; infant formulas, follow-on milks, baby food

Thus proposed reasons for increasing reliance on UPF include erosion of home food preparation skills and infrequent use of these skills. [23, 24] Adults in developed countries are spending less time on home food preparation, [25, 26] but there is little clear evidence for erosion of skills. For example, 89% of adults in the UK report being able to cook a main dish from basic ingredients. [27].

There is growing evidence that greater consumption of food prepared at home is associated with healthier diets. [28] However, as far as we are aware, no previous study has explored the relationship of home food preparation skills and behaviour with consumption of UPF in particular. We examined this relationship in UK adults. We hypothesised that those with better home food preparations skills and those who prepared food at home more frequently would consume a diet less reliant on UPF.

## Methods

We conducted a cross-sectional analysis of data from the UK National Diet and Nutrition Survey (NDNS) 2008–09. [29] This study is reported according to the STROBE-nut checklist. [30].

### Data source

NDNS is an annual, cross-sectional survey collecting detailed dietary data and information on a range of other personal and household characteristics and food behaviours. In 2008–09 a series of questions on home food preparation skills and behaviours were included.

Household selection for NDNS takes place using multi-stage probability sampling. Firstly, a random sample of small geographical areas are selected to allow more efficient, geographically focused, data collection. Private addresses are then randomly selected within these areas from the Postcode Address File (a list of all addresses in the UK). If more than one household lives at a particular address, one is randomly selected for inclusion. Up to one adult (aged 19 years or older) and one child (aged 1.5–18 years) from each household is then randomly selected to take part. Households are first contacted by letter a few days before an interviewer visits. Data collection involves an in-person interview covering personal and household characteristics. In 2008–09 information on cooking skills and behaviours was also collected during this interview.

Following the interview an estimated four-day food diary with portion sizes based on common household measurements is then completed. [29] Dietary supplements were not included in our analyses. Those who completed three or four of the food diary days were thanked with shopping vouchers with a value of £30 (approx. €41.20, US$46.60). Data collection takes place throughout the year and diary days are selected to ensure balanced representation of all days of the week.

In 2008–09, NDNS reported that 89% (raw n not available) of households eligible for inclusion agreed to take part. Usable food diaries (three or four completed days) were collected from at least one person in 64% of eligible households. Overall 55% of those selected to take part completed usable diaries. [29].

### Inclusion criteria

All individuals aged at least 19 years at the time of participation, who completed three or four days of the food diary, and did not report any health problems limiting or preventing them from cooking were included in the analyses. Those younger than 19 years were excluded as they were not asked the questions on home food preparation skills and behaviour.

### Variables of interest

#### Home food preparation skills and behaviour

Home food preparation skills and behaviour were measured using four variables: confidence with eight cooking techniques, confidence with cooking 10 foods, ability to prepare different dishes without help, and frequency of cooking main meals (see Table 2). The provenance of these questions is unclear and we are not aware of any published data on reliability and validity. However, the same or similar questions have been used in previously. [27, 31] As around half of participants reported confidence with all eight techniques or all 10 foods, these variables were dichotomised into confidence or not with all eight techniques or all 10 foods. As 89% of respondents answered “Yes, with no help at all” to the first three types of dish, participants were dichotomised into those who were and were not able to bake a cake or biscuits with no help at all. To maintain comparability with previous data [27, 31] we dichotomised answers concerning frequency of preparing a main meal into most days (five days of the week or more) and less often.

**Table 2:** Questions used to measure home food preparation skills and behaviours

Measure of home food preparation skills and behaviours	Questions	Response options
Confidence in using eight cooking techniques	Which, if any, of the following cooking techniques do you feel confident about using?	Boiling, steaming or poaching, frying, stir frying, grilling, oven-baking or roasting, stewing, braising, or casseroling, microwaving
Confidence in cooking ten foods	Which, if any, of the following foods do you feel confident about cooking?	Red meat, chicken, white fish (cod, haddock, plaice), oily fish (herring mackerel, salmon), pulses (such as split peas and lentils), dry pasta, rice (savoury), potatoes (not chips), green vegetables (cabbage, spinach, broccoli), root vegetables (carrots, parsnips)
Ability to prepare four different types of dish	Would you be able to make the following foods and dishes from beginning to end: convenience foods and ready meals (e.g. frozen pizza, pre-packaged curry & rice), a complete meal from ready-made ingredients (e.g. ready-made sauces and pasta to make spaghetti Bolognese), a main dish from basic ingredients (raw potatoes, raw meat, onions etc.), possibly following a recipe (e.g. shepherd’s pie, curry), a cake or biscuits from basic ingredients (flour, milk, eggs, etc.), possibly following a recipe	No, not at all, Yes, with a lot of help, Yes, with a little help, Yes, with no help at all
Frequency of preparing main meals	How often do you prepare a main meal for yourself or others?	Never, only for special occasions, less than once a week, one or two days a week, some days (3–4 a week), most days (5–6 a week), every day

#### Consumption of ultra-processed foods

Percentage of total energy intake obtained from UPF was calculated as previously. [2] In brief, firstly all foods in the NDNS nutrient databank were coded according to their degree of processing using the classification described in Table 1. [1] These codes were then merged with individual food diary data to determine the percentage of dietary energy each individual consumed from UPFs.

#### Other co-variates

Co-variates included were gender, age, occupational social group, and household composition (measured in terms of whether or not other adults or children were present in the household). Occupational social group was measured using the National Statistics Socio-Economic Classification (NS-SEC) of the highest household earner. The full eight-level classification was collapsed into three groups for analysis: routine and manual, intermediate, or managerial and professional occupations. [32] Where no member of the household was currently employed classification was made based on the last main jobs of household members. These co-variates were chosen as there is evidence that they are associated with home food preparation skills and behaviour, [27, 33] and UPF consumption. [2, 15].

#### Analysis

We used linear regression to explore associations between home food preparation skills and behaviour (‘exposure’ variables) and percentage of dietary energy from UPF (‘outcome’ variable). Separate models were run for each measure of home food preparation skills and behaviour (confidence with techniques, confidence with foods, ability to bake a cake or biscuits without help, and frequency of cooking). In all cases both unadjusted and fully adjusted (adjusted for sex, age, occupational social group, other adults in house, and children in household) models were run. As described above, all measures of home food preparation skills and behaviour were highly skewed and so were dichotomised in analyses. Throughout, age was entered as a continuous variable, occupational social class as an ordinal variable, and other adults and children in the household as binary variables.

## Results

A total of 548 adults aged 19 years or older completed three or four days of the NDNS food diary in 2008–09. Of these, 39 (7.1%) reported health problems limiting or preventing them from cooking. This left 509 meeting the inclusion criteria and included in the analysis. There were no other missing data.

The characteristics of individuals included in the analysis are shown in Table 3. There were more female than male individuals, individuals were relatively evenly distributed across the age spectrum, and were least likely to be in the intermediate occupational social group. Around two-thirds of individuals lived with other adults and one-third lived with children. Around half of individuals were confident with all eight techniques or all 10 foods, nearly three-quarters were able to bake cakes or biscuits without help, and more than two-thirds cooked a main meal for themselves or others on five or more days per week. Mean (SD) percentage of dietary energy obtained by individuals from UPF was 51.3% (13.1).

**Table 3:** Characteristics of individuals included in the analyses

Variable	Level	*n* (%); (*N* = 509)
Sex	Male	221 (43.4)
	Female	288 (56.6)
Age group (years)	19–29	84 (16.5)
	30–39	102 (20.0)
	40–49	84 (16.5)
	50–59	100 (19.7)
	60–69	76 (14.9)
	70+	63 (12.4)
NS-SEC	Professional & managerial	204 (40.1)
	Intermediate	101 (19.8)
	Routine & manual	204 (40.1)
Household composition	Participant lives with other adults	346 (68.0)
	Participant lives with children	184 (36.2)
Home food preparation skills and behaviour	Confident with all 8 techniques	273 (53.6)
	Confident will all 10 foods	261 (51.3)
	Able to bake cake/biscuits without help	364 (72.5)
	Cook main meal 5+ times per week	347 (68.2)

*NS-SEC* National Statistics Socio-economic Classification

Unadjusted associations between individuals’ home food preparation skills and behaviours and their consumption of UPF are shown in Table 4; adjusted models are shown in Table 5. In adjusted models being confident with all 10 foods, being able to bake cakes or biscuits without help, and cooking a main meal at least five days a week were statistically associated with consuming a lower percentage of dietary energy from UPF. Confidence with all 8 cooking techniques was not associated with consumption of UPF. The only other significant correlate of percentage of dietary energy obtained from UPF was age – older individuals consumed a lower percentage of dietary energy from UPF.

**Table 4:** Unadjusted associations between individuals’ home food preparation skills and behaviours and percentage of dietary energy from ultra-processed foods (*n* = 509)*

Cooking variable	Unadjusted regression coefficient (95% confidence intervals)
Confident with all 8 techniques (vs not)	−1.86 (−4.14 to 0.42)
Confident with all 10 foods (vs not)	−4.29 (−6.55 to −2.04)
Able to bake cake/biscuits without help (vs unable)	−2.49 (−5.05 to 0.07)
Cook main meal 5+ time per week (vs less often)	−2.70 (−5.14 to −0.26)

*Each row represents a separate model

**Table 5:** Adjusted associations between individuals’ home food preparation skills and behaviours and percentage of dietary energy from ultra-processed foods (*n* = 509)*

Variable	Adjusted regression coefficient (95% confidence intervals)			
	Confident with all 8 techniques	Confident with all 10 foods	Can bake cake or biscuits without help	Cook main meal 5+ time per week
Home food preparation skill or behaviour	−1.56 (−3.87 to 0.75)	−3.76 (−6.02 to −1.50)	−3.87 (−6.62 to −1.12)	−2.84 (−5.43 to −0.24)
Sex	1.31 (−0.99 to 3.62)	1.51 (−0.77 to 3.79)	2.71 (0.20 to 5.22)	2.09 (−0.36 to 4.53)
Age	−0.16 (−0.24 to −0.09)	−0.16 (−0.23 to −0.09)	−0.17 (−0.24 to −0.09)	−0.16 (−0.23 to −0.09)
Other adults in household	0.45 (−2.07 to 2.97)	0.50 (−1.99 to 3.00)	0.46 (−2.05 to 2.97)	0.18 (02.35 to 2.70)
Children in household	0.54 (−2.18 to 3.26)	0.25 (−2.43 to 2.92)	0.64 (−2.05 to 3.33)	0.31 (−2.39 to 3.00)
NS-SEC (intermediate vs managerial & professional)	−1.05 (−4.11 to 2.02)	−1.20 (−4.24 to 1.84)	−1.25 (−4.30 to 1.80)	−0.83 (−3.89 to 2.23)
NS-SEC (routine & manual vs managerial & professional)	1.52 (−1.02 to 4.07)	1.06 (−1.46 to 3.59)	1.63 (−0.87 to 4.13)	1.85 (−0.63 to 4.35)

*NS-SEC* National Statistics Socio-economic Classification

*Each column represents a separate model with adjustment for all variables listed

## Discussion

### Summary of results

This is the first exploration of the association between home food preparation skills and behaviour, and consumption of UPF that we are aware of. In partial support of our hypothesis we found that some markers of individuals’ own home food preparation skills and behaviour were significantly associated with UPF consumption. Where associations were found, greater home food preparation skills or frequency was associated with lower consumption of UPF.

### Interpretation of results

Where significant associations were present, we found that those who were more confident or prepared food more frequently consumed around 3–4 fewer percentage points of energy from UPF than others. With a mean daily energy intake in the sample of 7894 kJ (1887 kcal) (data not shown), this difference represents around 237–316 fewer kJ (57-75 kcal) from UPFs per day; or around 40–50% of a 330 ml can of regular Coca-cola. This difference is not insubstantial and reflects previous findings of relationships between both better home cooking skills and greater frequency of consuming home cooked food, and dietary quality. [28, 34].

Our results were not entirely consistent. Confidence with all eight techniques was not associated with consumption of UPF. Perhaps somewhat un-intuitively, it is possible that confidence with all eight techniques is the measure that is least related to practical use of home food preparation skills. For instance confidence with preparing a range of foods, being able to bake a cake or biscuits, and preparing a main meal frequently might all be expected to reflect individuals’ applied use of home food preparation skills. In contrast, confidence with all eight techniques may reflect more theoretical knowledge. Previous authors have highlighted the limitations of existing conceptualisations and measures of home cooking skills and behaviours. [35] Further work is required to develop valid and reliable measures of clear concepts. [36, 37].

The only other consistent correlate of individuals’ UPF consumption that we found was age. In all cases there was an inverse association with older individuals tending to consume a lower percentage of energy from UPF. Similar patterns have been previously described in relation to UPF consumption in particular, [2] and ‘healthier’ diets in general. [29] Further work will be required to determine if this a true age effect or a cohort effect.

### Implications of results for research, policy and practice

Our finding that some, but not all, measures of home food preparation skills and behaviours are associated with UPF consumption reiterate the complexity of home food preparation. [35] Our analyses also highlight the poor current conceptualisation of what home food preparation skills and behaviours are, [35] what aspects of them matter for diet and health, [28] and the absence of valid and reliable measures of these skills and behaviours. [36, 37] Despite using the most comprehensive food and diet dataset currently available in the UK, these issues all limit the interpretations we can make. Researchers need to make progress on conceptualisation and operationalisation of home food preparation skills and behaviour, and how these may influence dietary quality and health as a matter of urgency.

Further research is also needed to confirm our results in longitudinal settings and so increase confidence that the associations reported here are causal. If this is confirmed our results suggest that providing individuals with practical home food preparation skills and encouraging them to use these on most days of the week may be one method to decrease consumption of UPFs.

### Limitations of methods

Our study is cross-sectional and the associations reported should not be interpreted as causative. Further longitudinal research is necessary to determine the direction of any causation between home food preparation skills and behaviour and consumption of UPF.

Although NDNS invites a population-representative sample, the achieved sample is not necessarily representative due to selective non-response. [29] However, the sample included in our analysis represents the full diversity of the UK population in terms of age, gender and socio-economic characteristics meaning the associations we found are likely to be generalizable to the UK. Given international differences in food preparation and consumption behaviours [38, 39] our findings may not be more widely generalizable.

The data we used was entirely self-reported and may be subject to social desirability bias. Whilst food diaries are recognised to be one of the most comprehensive methods of assessing dietary intake, [40] selective under-reporting of some foods (particularly less healthy foods) occurs. [41, 42] The extent and nature of this may vary between population sub-groups. [41, 42] There may also be social pressures to over-report home food preparation skills and behaviour. Variations in interpretation of the questions used to assess home food preparation skills and behaviour may have introduced further error or bias. The provenance of the questions on home food preparation skills and behaviour is unclear and we are not aware of any published assessments of validity or reliability. There are few established, agreed and validated measures of cooking skills. [37] This is partly related to the poor conceptualisation of home cooking. [35] It is difficult to predict what the influence of these potential biases may have been on the results reported. Given how difficult it is to assess selective under-reporting, we did not correct for it.

The data used are now 7–8 years old and may not reflect the current situation in the UK. However, they are the most recent and comprehensive data from the UK we are aware of on this topic.

## Conclusion

In UK adults, better home food preparation skills and more frequent use of these skills tended to be cross-sectionally associated with lower UPF consumption. Further work is required to conceptualise and operationalise home food preparation skills and behaviours. Greater encouragement of these skills may help reduce reliance on UPF.
